# *Bacillus subtilis* Inhibits *Vibrio natriegens*-Induced Corrosion via Biomineralization in Seawater

**DOI:** 10.3389/fmicb.2019.01111

**Published:** 2019-05-21

**Authors:** Zhangwei Guo, Shuai Pan, Tao Liu, Qianyu Zhao, Yanan Wang, Na Guo, Xueting Chang, Tong Liu, Yaohua Dong, Yansheng Yin

**Affiliations:** ^1^College of Ocean Science and Engineering, Shanghai Maritime University, Shanghai, China; ^2^College of Materials Science and Engineering, Qingdao University of Science and Technology, Qingdao, China

**Keywords:** low-alloyed steel, *Bacillus subtilis*, *Vibrio natriegens*, seawater, corrosion inhibition, biomineralization

## Abstract

The marine bacterium, *Vibrio natriegens*, grows quickly in a marine environment and can significantly accelerate the corrosion of steel materials. Here, we present an approach to inhibit *V. natriegens*-induced corrosion by biomineralization. The corrosion of steel is mitigated in seawater via the formation of a biomineralized film induced by *Bacillus subtilis*. The film is composed of extracellular polymeric substances (EPS) and calcite, exhibiting stable anti-corrosion activity. The microbial diversity and medium chemistry tests demonstrated that the inhibition of *V. natriegens* growth by *B. subtilis* was essential for the formation of the biomineralized film.

## Introduction

When steel materials are exposed to a marine environment, galvanic corrosion coupled with microbiologically influenced corrosion (MIC) will inevitably occur, resulting in tremendous economic loss and security risk (Li et al., 2015). Approaches to retard steel corrosion include cathodic protection (Bagherzadeh and Mousavinejad, 2012), coatings (Shchukin et al., 2006; Andreeva et al., 2008), and corrosion inhibitors. While these approaches are effective, they are limited by high cost, maintenance difficulties, environmental pollution, and short service time (Banerjee et al., 2012; Nkuzinna et al., 2014). In our previous study, we demonstrated that some biofilms are able to provide an environmentally friendly, stable, and lasting biomineralized film to inhibit corrosion in a marine environment (Liu et al., 2019). The film is composed of extracellular polymeric substances (EPS) and calcium carbonate with a calcite structure, exhibiting a stable and self-healing anti-corrosion activity. Bacterial metabolism produces large amounts of CO_2_. The precipitation of CaCO_3_ is mainly related to the release of CO_2_ from bacteria, resulting in supersaturating with respect to calcium carbonate. In this process, abundant EPS around bacteria provides nucleation site for precipitation of CaCO_3_.

However, the natural environment includes diverse microorganisms, may hinder this application. Specifically, bacteria that compete for growth in a natural marine environment might affect the biomineralization (Tan et al., 2018). Therefore, further investigation into biofilm-induced mineralization within a competitive bacterial medium is critical for its practical application.

The marine bacterium *Vibrio natriegens* is widely distributed in the ocean, which was first reported by Payne in 1958. *V. natriegens* grows faster than other organisms in the marine environment (Aiyar et al., 2002; Cheng et al., 2009; Mullenger and Gill, 2010; Weinstock et al., 2016; Hoffart et al., 2017). As a result, *V. natriegens* is typically one of the early colonizers in biofilm communities (Nadia et al., 1991). Moreover, *V. natriegens* is able to acquire electrons during the process of N_2_-fixation, which further accelerates corrosion of substrate materials under certain conditions (Angell et al., 1995; Coyer et al., 1996; Little et al., 2008; Cheng et al., 2009; Yin et al., 2009).

Herein, we find that the MIC by *V. natriegens* can be inhibited by the addition of *Bacillus subtilis* in the marine broth. Interestingly, co-culture of *V. natriegens* and *B. subtilis* resulted in a distinguishing effect on the corrosion of steel. Morphological and electrochemical analyses showed that *B. subtilis* provided anti-corrosion protection for steel in the presence of *V. natriegens*. The biomineralized film was not influenced by the competitive growth of *V. natriegens*. The chemical composition of the solution showed that a decrease in magnesium (Mg^2+^) and an increase in pH occurred during the biomineralization process induced by *B. subtilis*, which contributed to the inhibition of *V. natriegens* growth and metabolism. Therefore, this study indicated that the biomineralized film induced by *B. subtilis* biofilm formation is a promising approach for reducing the corrosion of steel in a complex bacterial environment.

## Materials and Methods

### Materials and Specimens

The low-alloyed steel plates were supplied by Baosteel Inc., (China). The chemical composition of steel is: 1.52 wt% Mn, 0.70 wt% Ni, 0.04 wt% Al, 0.15 wt% Cr, 0.05 wt% C, 0.40 wt% Mo, 97.14 wt% Fe. The coupons were covered by the epoxy resin, leaving a 10 mm × 10 mm working area, and then polished from 200 to 800 grit, washed with absolute ethyl alcohol, deionized water sequentially. Lastly, they were N_2_-dried and sterilized by ultraviolet (UV) light.

### Bacterium Culture

The bacterial strains, *B. subtilis* and *V. natriegens*, were isolated from the East China Sea. The two bacterial strains were cultivated in a 2216E medium on a shaker at 120 rpm at 37°C. 2216E medium: 5 g peptone, 1 g yeast extract, and 0.01 g ferric phosphate (Sinopharm Chemical Reagent Co., Ltd., China) was added into 1 L seawater, then sterilized at 121°C for 15 min. The coupons were immersed in 1-L containers with 500 mL of 2216E medium. Two hundred microliters of an overnight culture of *V. natriegens* strain was used to inoculate the first container. To investigate the effect of *B. subtilis* on corrosion by *V. natriegens*, one hundred microliters of each strain was then used to inoculate the second container. The two containers were then cultured at 120 rpm for 13 days at 37°C. The relative abundance was measured using bacterial diversity analysis at Majorbio (Shanghai, China). The primers sequence was: 338F: 5′-ACTCCTACGGGAGGCAGCAG-3′, 806R: 5′- GGACTACHVGGGTWTCTAAT-3′. The V3+V4 region of the bacterial 16S rRNA gene was amplified by the above primers. PCR reaction conditions was: 95°C 3 min; 95°C 30 s, 55°C 30 s, 72°C 45 s, 30 cycles; 72°C 10 min; 10°C 10 min. PCR reaction system was: 5 × PCR buffer (with Mg^2+^) 4 μL; 2.5 mmol/L dNTP 2 μL; 5 μmol/L P1 (338F) 0.8 μL; 5 μmol/L P2 (806R) 0.8 μL; 5 U/μL Taq enzyme 0.4 μL; DNA template 2 μL; ddH_2_O 10 μL. It was then sequenced on an Illumina MiSeq PE250/PE300. The sequencing work was completed by Majorbio (Shanghai, China). Relative abundance was obtained from rank-abundance function on www.i-sanger.com.

### Morphological and Composition Characterization

After 2 weeks of growth, the coupons were washed gently with a phosphate buffer solution (PBS, pH = 7.4) and then immersed in 2.5% glutaraldehyde solution (Sinopharm Chemical Reagent Co., Ltd., China) for 15 min. Subsequently, the coupons with biofilms were dehydrated by gradient ethanol solution (30, 50, 60, 70, 80, 90, and 100%) sequentially for 10 min and dried with nitrogen gas. The morphology of the covered surface was characterized by a scanning electron microscope (SEM) with an energy dispersive spectrometer (JEOL JSM-7500F, Japan). The biofilm and corrosion products were removed using the NACE Recommended Method 0775-1999 protocol (NACE, 1999). The coupons were immersed sequentially in a solution of dibutyl thiourea hydrochloride, sodium bicarbonate (NaHCO_3_) solution (Sinopharm Chemical Reagent Co., Ltd., China), and water for 2 min each. The morphology of the coupons was observed by a 3-D optical profilometer (Bruker Contour GT, Germany).

X-ray diffraction (XRD; X’Pert PRO XRD, PANalytical, Almelo, Netherlands) was applied to investigate the phase identification of the film on the coupons (40 kV, 10 mA, Cu-Kα radiation source, 0.26°/s scanning rate, 2𝜃 degree of 20–90°). Furthermore, the steel coupons covered with biofilms were analyzed by X-ray photoelectron spectroscopy (XPS; Kratos, AXIS Ultra DLD, England; 200 μm spot size, 0–1200 eV, 45° emission angle). High-resolution spectra of C 1s and O 1s scans were obtained. Experiments were performed with three random tests on the different scanning areas, and only representative images were shown.

### Corrosion Measurements

The electrochemical methods, such as open-circuit potential (OCP), linear polarization (LPR), and electrochemical impedance spectroscopy (EIS), were carried out using an electrochemical workstation (Ivium, Eindhoven, Netherlands). The steel coupons were working electrodes. A saturated calomel electrode (SCE) and a platinum (Pt) sheet were the reference electrode and the counter electrode, respectively. Prior to the measurements, the OCP of the steel coupons was monitored for at least 60 min in order to reach a steady state. The LPR measurement was obtained at a scan rate of 0.125 mV s^-1^ in the range from -5 to 5 mV versus the OCP, with a sampling frequency of 1 Hz (Xu et al., 2018). The EIS measurement was measured with a frequency range between 1 × 10^-2^ and 1 × 10^5^ Hz and ± 10 mV amplitude. The impedance data were analyzed by ZSimpWin. The corrosion rate was obtained from weight loss tests using the following equation:

$$\begin{array}{c} V_{\mathrm{corr}}{(mm\cdot y}^{-}{}^{1})={[87600 \times \Delta m(g)]/\rho (g\cdot cm}^{-}{}^{3}{)A(cm}^{2})t(h). \end{array}$$

In addition, we used a local electrochemical impedance spectroscopy (LEIS) workstation (Bio-logic M470, Seyssins, France) to study the local impedance map on the coupon surface. A carbon rod was used as the counter electrode with a SCE as the reference electrode. The surface of coupon was the working electrode. There was a 100-μm distance between the working electrode and the Pt probe tip. The LEIS maps were measured with 10 μA current amplitude and 10 Hz frequency. We chose the 10 Hz single frequency because the curve of impedance vs. frequency was at the logarithm frequency interval of 10 Hz frequency, which is beneficial to the stable testing and resistance to the disturbance (Liu J. et al., 2018; Ma et al., 2018).

### Chemical Analysis of Two Containers

The content of Ca^2+^, Mg^2+^, and NH_4_^+^ was measured to investigate N_2_-fixation of *V. natriegens* in two containers. pH was also obtained in this process. The Ca and Mg concentration in the seawater was investigated by ICP in first 5 days. NH_4_^+^ in medium was determined by ion chromatography (Thomas et al., 2002). Three replicates were measured in all cases, and the background correction was obtained using blank samples.

## Results

**FIGURE 1:**
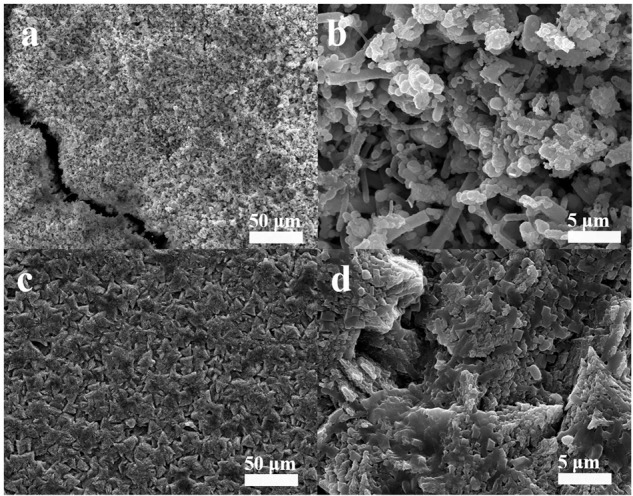
Scanning electron microscope views of the morphology of the biofilm of **(a,b)** only *V. natriegens* after 13 days and **(c,d)** co-cultures of *B. subtilis* and *V. natriegens*. Image of two different magnitudes are shown in the **(a,c)** left and **(b,d)** right panel.

Scanning electron microscope was used to investigate the surface morphology of the coupons from co-cultures of *B. subtilis* and *V. natriegens* in marine broth after 13 days. The control culture contained only the *V. natriegens* strain. Figure 1a,b illustrate the rough, cracked, and heterogeneous corrosion products that formed on the steel surface in the control medium. The iron oxide (Fe_3_O_4_) formed in a petal-like manner on the surface of the steel coupons. In contrast, only triangular-shaped minerals mineral precipitation was present on the steel in the medium containing *B. subtilis* and *V. natriegens* at day 13 (Figure 1c,d). The biomineralized film was homogeneous and compact, demonstrating a potential to protect steel coupons. Obviously, the biomineralized film that formed on the coupons was not influenced by the competitive growth of *V. natriegens*. The energy dispersive spectrometer measured the film composition on day 13. As shown in Supplementary Figure S1a, the amount of C, O, Mg, and Ca on the steel coupons in the control medium was 3.4, 24.1, 0.3, and 5.3 wt%, respectively. In contrast, the amount of C, O, Mg, and Ca on the steel coupons in the presence of both *B. subtilis* and *V. natriegens* was 13.3, 36.5, 4.0, and 44.5 wt%, respectively. These results indicate that the biomineralized film formed on the steel in the competitive bacterial environment.

**FIGURE 2:**
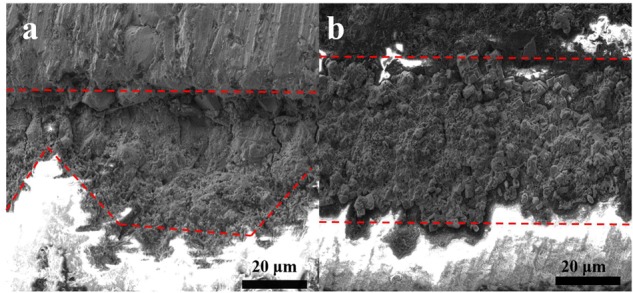
Cross-section views of the biofilm of **(a)** only *V. natriegens* and **(b)** co-cultures of *B. subtilis* and *V. natriegens* after 13 days.

Cross-sectional SEM images were analyzed in order to investigate the thickness of the biomineralized film and the film-substrate interface. The average thickness of the corrosion product film on the steel coupon in the control culture was approximately 29 μm, as shown in Figure 2a. Figure 2b shows that the thickness of the biomineralization film, which formed on the steel in the co-culture, was approximately 44 μm. The biomineralization film and steel interface was compact in the presence of *B. subtilis* and *V. natriegens*. In contrast, in the presence of only *V. natriegens*, the corrosion product film was relatively loose with obvious cracks, which are unable to resist the penetration of chloride ions to the steel.

**FIGURE 3:**
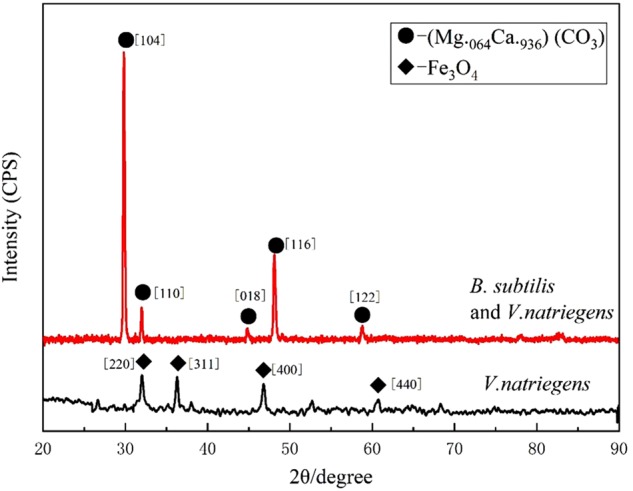
X-ray diffraction spectrum measured on the coupon immersed in medium with only *V. natriegens* (black line), and in the co-cultures of *B. subtilis* and *V. natriegens* (red line), respectively.

**FIGURE 4:**
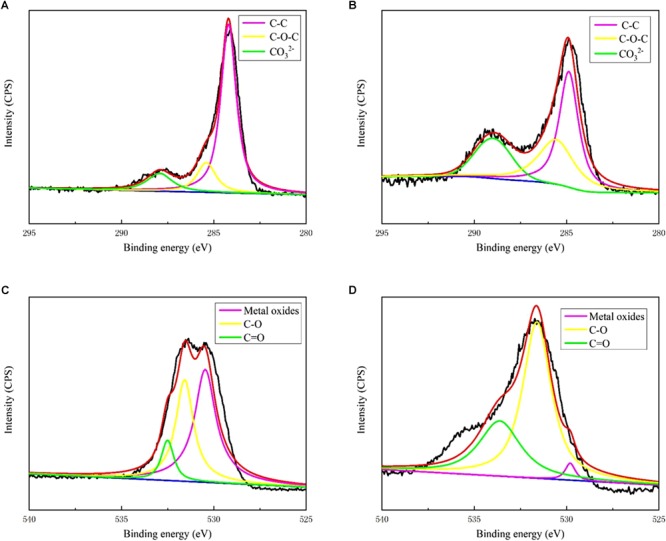
X-ray photoelectron spectroscopy spectra of C 1s for biofilm of **(A)** only *V. natriegens* and **(B)** co-cultures of *B. subtilis* and *V. natriegens*; O 1s for biofilm of **(C)** only *V. natriegens* and **(D)** co-cultures of *V. natriegens* and *B. subtilis*.

Figure 3 shows the XRD patterns of the steel surfaces after 13 days. In the medium containing *B. subtilis* and *V. natriegens*, calcium and magnesium carbonate were the main components of the biomineralization film, which was consistent with our previous study (Liu T. et al., 2018). Fe_3_O_4_ was the main component of the corrosion products film in the medium with *V. natriegens*. High-resolution XPS of C 1s and O 1s from the coupons is shown in Figure 4. In the C 1s spectrum, the peaks at 284.6, 285.4, and 288.4 eV are attributed to the binding energies of C-C, C-O-C, and CO_3_^2-^ bonds, respectively (Figure 4A,B). According to the XRD analysis, the carbonate was calcium magnesium carbonate, while the C-C and C-O-C signals were attributable to the biofilm on the coupon (Liu et al., 2016). Table 1 shows that the film had a higher carbonate content (41%) in the presence of *B. subtilis* and *V. natriegens*, whereas the amount of carbonate was only 10% in the medium with *V. natriegens*, which was consistent with the XRD results. In the O 1s spectrum (Figure 4C,D), the peaks of 529.8, 531.6, and 533.6 eV were caused by the combined energy of the metal oxide (Fe_3_O_4_), C-O, and C=O bonds, respectively (Gauzzi et al., 1990). The data in Table 1 indicate that the amount of metal oxide (529.8 eV) was 52 and 16% in the control medium and the co-cultures medium, respectively, suggesting that the corrosion products (Fe_3_O_4_) decreased due to the formation of a biomineralized film.

**Table 1:** Characterization of XPS spectra of C 1s and O 1s on the different surfaces.

		Binding	Proposed
Valence state	Substrate	energy (eV)	components	%
C 1s	*V. natriegens*	284.6	C-C	74.79
		285.4	C-O-C	14.91
		288.4	CO_3_^2-^	10.29
	*B. subtilis* and *V. natriegens*	284.6	C-C	22.92
		285.4	C-O-C	35.00
		288.4	CO_3_^2-^	41.08
O 1s	*V. natriegens*	529.8	Metal oxides	52.48
		531.6	C-O	37.64
		533.6	C=O	09.86
	*B. subtilis* and *V. natriegens*	529.8	Metal oxides	15.89
		531.6	C-O	62.66
		533.6	C=O	21.45

**FIGURE 5:**
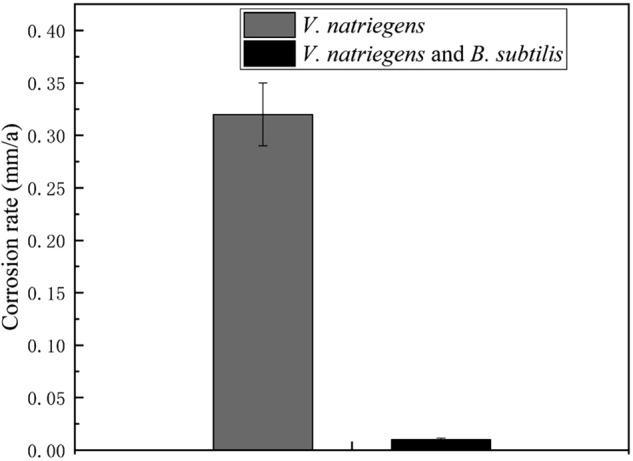
Corrosion rate of coupons after 30 days of exposure in different media. The errors shown for each data is the standard deviation obtained from five sets of measurements.

In our previous study, we found that the biomineralized film induced by *B. subtilis* exhibited excellent corrosion resistance. However, the experimental medium only contained the *B. subtilis* strain, and the protection of the biomineralized film in a competitive bacterial environment was not explored. Figure 5 shows the corrosion rates of the steel coupons in the co-cultures medium and control medium. The corrosion rate in the presence of *V. natriegens* was 0.32 mm/a corrosion, which was 32-fold higher than the corrosion rate in the presence of *B. subtilis* and *V. natriegens* (0.01 mm/a). These results indicate that *B. subtilis* effectively inhibited the uniform *V. natriegens*-induced corrosion of steel via biomineralization.

**FIGURE 6:**
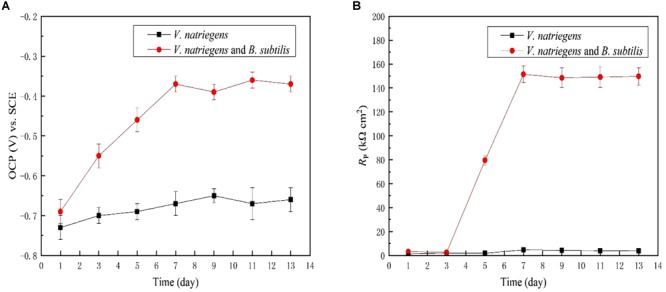
Time dependence for **(A)** OCP and **(B)** *R_p_* for the coupons immersed in different media.

Figure 6a shows the time dependence of the OCP of the steel coupons for 13 days. In the medium with only *V. natriegens*, the OCP of the steel coupon fluctuated at approximately -0.69 V (SCE) during the entire immersion period. However, in the presence of *V. natriegens* and *B. subtilis*, the OCP moved positively to -0.39 V (SCE) on day 13, indicating the inhibition of an anodic reaction by the biomineralized film. LPR was utilized to evaluate the *R_p_* value corresponding to the corrosion rate based on a small overpotential. Figure 6b shows that the *R_p_* value of the steel coupon in the co-cultures medium was relatively higher than the value in the control medium, consistent with the results of the weight loss test.

**FIGURE 7:**
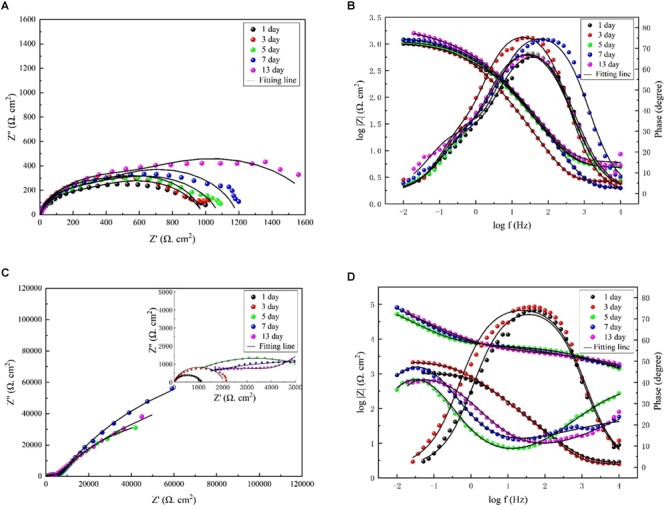
Nyquist and Bode diagrams measured on the coupons immersed in medium **(A,B)** with only *V. natriegens*, and **(C,D)** the co-cultures of *B. subtilis* and *V. natriegens*, respectively.

Additionally, EIS was applied to evaluate the electrochemical process of the interface of the medium, film, and steel. Figure 7A shows that two time constants appeared on the Nyquist diagram in the marine broth with *V. natriegens* at day 1. This could be attributed to the rapid formation of the corrosion product film when the steel coupons were immersed in the medium. The equivalent circuit, i.e., *R_s_*(*Q_f_*(*R_f_*(*R_ct_Q_dl_*))), is shown in Supplementary Figure S2a. The fitted data (Table 2) indicated that the charge transfer resistance (*R_ct_*) increased from 262.4 to 758.3 Ω cm^2^ and then decreased to 318.3 Ω cm^2^ on day 7. On day 13, the *R_ct_* value increased to 1149.0 Ω cm^2^. The irregular changes of the *R_ct_* value with the immersion time resulted from the shedding and deposition of corrosion products. However, in the presence of both *V. natriegens* and *B. subtilis*, Nyquist diagrams exhibited rather different shapes during the immersion time. During the early stage (before day 7), one loop, which corresponded to one time constant in the Nyquist diagram, indicated that the mineral deposits were not well-formed (Supplementary Figure S2b). At day 9, two time constants were observed, indicating the formation of a compact biomineralized film on the steel coupons. The equivalent circuit, i.e., *R_s_*(*Q_f_*(*R_f_*(*R_ct_Q_dl_*))), is shown in Supplementary Figure S2c. The *R_f_* value that was attributed to the barrier effect of the biomineralized film increased from 1031 to 2.37 × 10^5^ Ω cm^2^. These results demonstrate that *B. subtilis* effectively protected the steel coupons from *V. natriegens*-induced corrosion.

**Table 2:** Electrochemical impedance parameters fitted from the measured impedance plots in Figure 7.

	*R*_s_ (Ω ⋅ cm^2^)	CPE1 (S Sec^∧^n/cm^∧^2)	*n*	*R*_ct_ (Ω ⋅ cm^2^)	CPE2 (S Sec^∧^n/cm^∧^2)	*n*	*R*_f_ (Ω ⋅ cm^2^)
*V. natriegens* (1 day)	4.648	0.000256	0.876	262.4	0.001046	0.781	719.4
*V. natriegens* (3 day)	2.557	0.001381	0.886	626.1	0.000383	0.913	349.7
*V. natriegens* (5 day)	4.710	0.000979	0.799	758.3	0.002892	0.876	319.0
*V. natriegens* (7 day)	1.841	0.000189	0.917	318.3	0.000844	0.851	871.1
*V. natriegens* (13 day)	5.776	0.001334	0.796	1149.0	0.000238	0.855	540.8
*B. subtilis* + *V. natriegens* (1 day)	2.633	0.000177	0.862	1031			
*B. subtilis* + *V. natriegens* (3 day)	2.254	0.000190	0.863	2053			
*B. subtilis* + *V. natriegens* (5 day)	66.380	0.000110	0.680	1.23 × 10^5^	0.000002	0.494	6290.0
*B. subtilis* + *V. natriegens* (7 day)	96.820	0.000076	0.704	2.33 × 10^5^	0.000016	0.315	7484.0
*B. subtilis* + *V. natriegens* (13 day)	0.001	0.000057	0.583	2.37 × 10^5^	0.000004	0.396	4447.0

**FIGURE 8:**
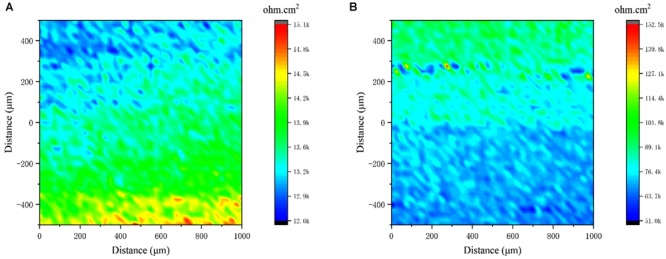
Local electrochemical impedance spectroscopy maps measured on the coupons immersed in medium with **(A)** only *V. natriegens*, and **(B)** co-cultures of *B. subtilis* and *V. natriegens* after 13 days.

Local electrochemical impedance spectroscopy measurements were carried out at a single frequency, which has been widely used for monitoring pitting corrosion or evaluating the homogeneity of coating/film due to its localized sensitivity. Figure 8 shows that the highest impedance value of the biomineralized film was approximately 1.1 × 10^5^ Ω cm^2^, whereas the value in the medium with only *V. natriegens* was approximately 1.4 × 10^4^ Ω cm^2^. The impedance value gradient on the biomineralized film was much smaller than the corrosion products film, suggesting that the biomineralized film with calcite structure formed uniformly on the steel coupons.

**FIGURE 9:**
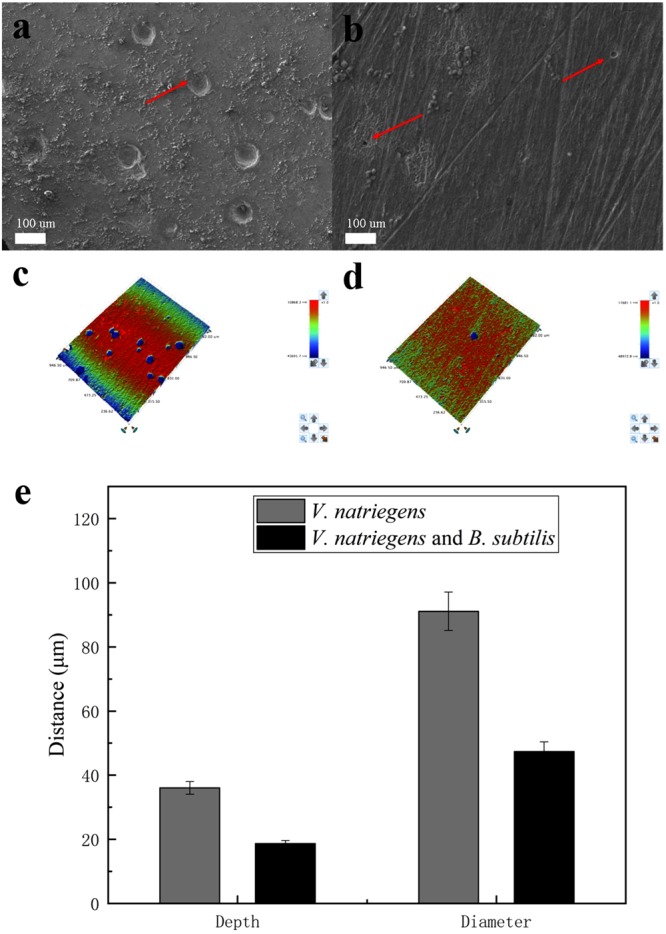
Scanning electron microscope and the optical profilometry images of the pit morphology on the coupon immersed in medium with **(a,c)** only *V. natriegens*, and **(b,d)** co-cultures of *B. subtilis* and *V. natriegens*, **(e)** and the derived topography.

Figure 9 shows the pit morphology of the steel coupons after removing the films. In the presence of *V. natriegens* and *B. subtilis*, the pits on the coupon were smaller and fewer than that in the medium with only *V. natriegens*. After analysis, the average depth of the pits was 36 μm, and the average diameter of the pits was 91 μm in the presence of *V. natriegens*. Both the number and the size of the pits decreased in the medium with the biomineralized film, with the pits measuring at 19 and 49 μm in depth and diameter.

**FIGURE 10:**
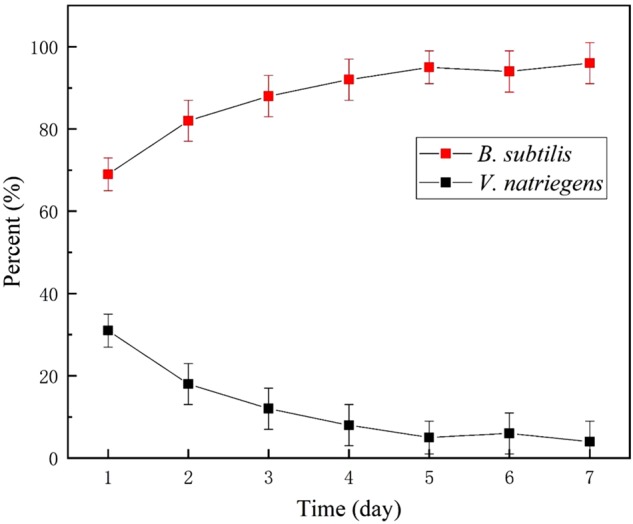
Relative content of *V. natriegens* and *B. subtilis* in co-cultures medium.

A microbial diversity test was utilized to investigate the relative content of the two bacteria as a function of time. Figure 10 shows that approximately 70% relative abundance was occupied by *B. subtilis* on day 2. However, at day 13, *B. subtilis* occupied the main content (96% relative abundance) of the medium, with only 4% relative abundance being identified as *V. natriegens*. This indicated that the growth of *V. natriegens* was inhibited by *B. subtilis*.

**FIGURE 11:**
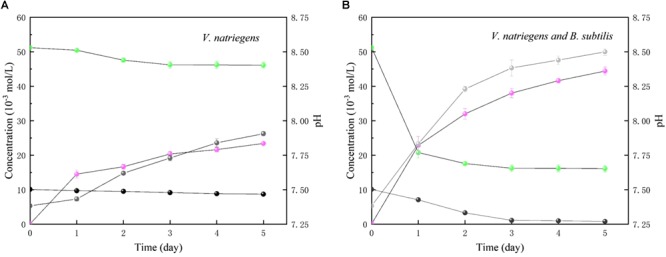
Time curses of pH (gray), soluble Ca (black), soluble Mg (green) and NH_4_^+^ (light magenta) in medium with **(A)** only *V. natriegens*, and **(B)** co-cultures of *B. subtilis* and *V. natriegens*.

In Figure 11, in the medium with single *V. natriegens*, only 10.1 and 14.2% of the initial soluble Ca and Mg was removed after 5 days. The concentrations of NH_4_^+^ increased from 0.11to 23.2 mmol/L. The value of pH increased from 7.37 to 7.90. While, in co-cultures of *B. subtilis* and *V. natriegens*, 30.4 and 61.4% of the initial soluble Ca and Mg was removed after first day, respectively (Figure 11b). After 5 days, 91.8 and 68.5% of the initial soluble Ca and Mg was removed. The concentrations of NH_4_^+^ increased from 0.12 to 43.4 mmol/L. The value of pH increased from 7.25 to 8.52.

## Discussion

Numerous studies have shown that corrosion of steel can be accelerated by the facultative anaerobic bacterium *V. natriegens*, which has the capability of N_2_-fixation. The process of biological N_2_-fixation is the reduction of N_2_ to ammonium:

(1) $$\begin{array}{c} N_{2}{+6e}^{-}{+6H}^{+}+nMg-ATP\to {2NH}_{3}+nMg-ADP+nPI, \end{array}$$

where the N_2_-fixation is a complex process that requires Mg^2+^, nitrogenases, and electron carriers. The hydrolysis of two Mg-ATP is accompanied by electron transfer. It has been shown that *V. natriegens* can accelerate the corrosion of steel by electron transfer and the nitrogenases associated with N_2_-fixation. Here, we present evidence that *B. subtilis* can inhibit *V. natriegens*-induced corrosion via biomineralization. Our previous work showed that *B. subtilis* could induce calcium carbonate precipitation on the surface of steel, decreasing the concentration of Ca^2+^ and Mg^2+^ in the medium. Furthermore, the pH in the medium inoculated with *V. natriegens* and *B. subtilis* was approximately 8.5, which is higher than the pH in the presence of only *V. natriegens* (approximately 7.9) (Figure 11). The increase in pH is attributed to the process by which microorganisms decompose nitrogenous organic compounds to produce ammonia via bacterial ammonification (Dannenmann et al., 2009). Microbial decomposition of organic nitrogen-containing compounds is the hydrolysis of macromolecules into small molecules by hydrolases secreted *in vitro* under aerobic or anaerobic conditions (Pan et al., 2018). As a microorganism with the capacity for ammonification, *B. subtilis* can decompose proteins and various nitrogen-containing organic substances to ammonium ions (NH_4_^+^) and bicarbonate (HCO_3_^-^), effectively, increasing the pH of the medium (Lipman, 1909; Hoffmann et al., 1998). Therefore, the N_2_-fixation process would be inhibited in the environment, which has a low concentration of Mg^2+^ and high pH value. In addition, the biodiversity experiment demonstrated that *B. subtilis* has a growth advantage in a competitive bacterial medium, even though *V. natriegens* was reported to have the shortest doubling time (9.8 min at 37°C). As a result, the initial biofilm structure is composed primarily of *B. subtilis* and not *V. natriegens*. Thus, calcium carbonate precipitation can be induced, thereby impeding the electron transfer between the anode and cathode on the steel surface. Consistent with these results, we found that the pitting corrosion was improved by the addition of *B. subtilis* via biomineralized film formation on the steel surface. The compact and hierarchical organic/inorganic film strongly contributed to the reduction of Cl^-^ permeation and electron transfer.

For the real application in the field, this method may be used in two approaches. First, we can use this method in a closed environment, e.g., ballast tank and pipeline, where have the relatively simple species of bacteria. When the corrosion occurs, the *B. subtilis* provides the anticorrosion activity for the steel as a corrosion inhibitor. Second, biomineralization can be used as a pretreatment method, leading to a high corrosion-resistance for the steel. Therefore, the culturing conditions that can be extended to the large scale treatment should be investigated carefully in the future.

## Conclusion

In this study, we evaluated the application of a biomineralized film in a competitive bacterial environment containing the corrosion-inducing bacterium. *B. subtilis* provided significant anti-corrosion protection for steel coupons in the presence of the corrosion-inducing bacteria, *V. natriegens*. Biomineralized film induced by *B. subtilis* was well formed on the steel surface in the co-cultures medium. The growth of *V. natriegens* was inhibited by *B. subtilis* due to the decrease in magnesium and the increase in pH within the medium as a result of biomineralization. Therefore, the results from this study show that *B. subtilis*-induced biomineralized film has the potential to be used in a real marine environment.

## Data Availability

No datasets were generated or analyzed for this study.

## Author Contributions

ZG, SP, QZ, YW, NG, XC, YD, and YY performed experiments and analyzed data. ToL provided software. ZG and TaL designed the experiments, analyzed data, and wrote the manuscript.

## Conflict of Interest Statement

The authors declare that the research was conducted in the absence of any commercial or financial relationships that could be construed as a potential conflict of interest.
