# Supplementary material for: Bacillus subtilis Inhibits Vibrio natriegens-Induced Corrosion via Biomineralization in Seawater
Source: Front Microbiol. 2019 May 21;10:1111. doi: 10.3389/fmicb.2019.01111 (PMC6536734; doi:10.3389/fmicb.2019.01111)
Supplement: Supplementary file 1 [file Data_Sheet_1.pdf]

Supplementary Material

Supplementary Figures

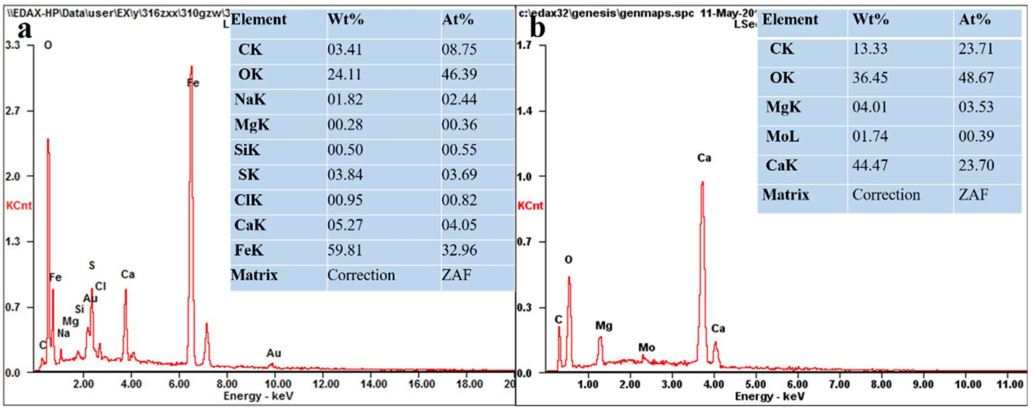

Supplementary Figure 1. EDS analysis of coupons in medium with (**a**) only *V. natriegens* and (**b**) co-cultures of *V. natriegens* and *B. subtilis*.

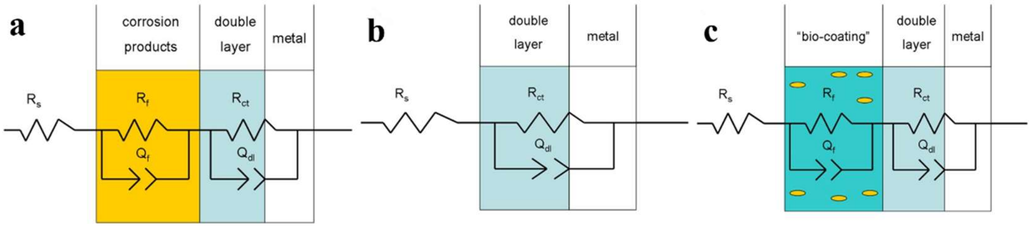

Supplementary Figure 2. Equivalent circuits used for simulate experimental impedance diagrams for the coupons in medium with (**a**) only *V. natriegens* and (**b**) co-cultures of *V. natriegens* and *B. subtilis* (before day 7) (**c**) co-cultures of *V. natriegens* and *B. subtilis* (day 9).
